# D6 protein kinase in root xylem benefiting resistance to *Fusarium* reveals infection and defense mechanisms in tung trees

**DOI:** 10.1038/s41438-021-00656-2

**Published:** 2021-11-01

**Authors:** Qiyan Zhang, Liwen Wu, Hengfu Yin, Zilong Xu, Yunxiao Zhao, Ming Gao, Hong Wu, Yicun Chen, Yangdong Wang

**Affiliations:** 1 State Key Laboratory of Tree Genetics and Breeding, Chinese Academy of Forestry, Beijing, 100091 China; 2 Research Institute of Subtropical Forestry, Chinese Academy of Forestry, Hangzhou, 311400 Zhejiang Province China

**Keywords:** Plant breeding, Biotic

## Abstract

*Fusarium oxysporum*, a global soil-borne pathogen, causes severe disease in various cultivated plants. The mechanism underlying infection and resistance remains largely elusive. *Vernicia fordii*, known as the tung tree, suffers from disease caused by *F. oxysporum* f. sp. *fordiis* (*Fof*-1), while its sister species *V. montana* displays high resistance to *Fof*-1. To investigate the process of infection and resistance ability, we demonstrated that *Fof*-1 can penetrate the epidermis of root hairs and then centripetally invade the cortex and phloem in both species. Furthermore, *Fof*-1 spread upwards through the root xylem in susceptible *V. fordii* trees, whereas it failed to infect the root xylem in resistant *V. montana* trees. We found that D6 PROTEIN KINASE LIKE 2 (*VmD6PKL2*) was specifically expressed in the lateral root xylem and was induced after *Fof-*1 infection in resistant trees. Transgenic analysis in *Arabidopsis* and tomato revealed that *VmD6PKL2* significantly enhanced resistance in both species, whereas the *d6pkl2* mutant displayed reduced resistance against *Fof*-1. Additionally, VmD6PKL2 was identified to interact directly with synaptotagmin (VmSYT3), which is specifically expressed in the root xylem and mediates the negative regulation responding to *Fof-*1. Our data suggested that *VmD6PKL2* could act as a resistance gene against *Fof*-1 through suppression of *VmSYT3*-mediated negative regulation in the lateral root xylem of the resistant species. These findings provide novel insight into *Fusarium* wilt resistance in plants.

## Introduction

*Fusarium oxysporum*, one of the top ten fungal plant pathogens^1^, is the causal agent of vascular wilt diseases of more than 120 plant species, provoking severe losses to crop production and the world economy^2,3^. Colonization of plants by *F. oxysporum* leads to necrosis of the infected tissues, collapse of vascular vessels, stunting, progressive wilting and defoliation of leaves, and decay of the plant^4^. As a ubiquitous soil-borne pathogen, *F. oxysporum* can survive in the soil for up to 30 years as durable chlamydospores^5^. At present, the mechanisms of infection and resistance are largely unknown; as a result, no efficient ways can completely control the outbreak and spread of *Fusarium* wilt.

*Vernicia* (Euphorbiaceae) species are promising industrial oil trees that produce tung oil from fruits. *Vernicia fordii* and *Vernicia montana* are the two main cultivated species. Compared to *V. montana*, *V. fordii* displays faster maturation periods and superior oil characteristics^6^. Therefore, oil refined from the seeds of *V. fordii* has been widely applied for the production of paints and coatings, inks, lubricants, synthetic rubber and biodiesel^7^. However, tung wilt disease caused by *F. oxysporum* f. sp. *fordiis* (*Fof*-1) has caused devastating damage to the growth and development of *V. fordii*. In contrast, *V. montana* shows notable resistance against *Fusarium* wilt disease^8^. However, the resistance mechanism of *Vernicia* against *Fusarium* remains largely unknown.

A comprehensive understanding of the interaction between plants and pathogenic *Fusarium* is crucial for elucidating the molecular basis of disease resistance and is invaluable for *Fusarium* wilt disease management^9^. This lack of a comprehensive understanding has driven a considerable amount of research on plant disease for many years^10^. However, knowledge about host-pathogen interactions in wilt disease is limited. Due to the complexity and potential variability of resistance to *F. oxysporum* among different plant species^4^, investigations toward the recognition of effective barriers that can limit pathogen invasion and the identification of novel resistance-related factors in forest trees should be urgently developed.

The *Vernicia* genus contains both resistant and susceptible species, which share high similarities in morphology, anatomy and karyotype. Therefore, it is an appreciated model for the investigation of the infection and resistance mechanism of *Fusarium* wilt disease. To reveal the detailed infection process of soil-born *Fusarium* in plants and the possible resistance mechanism of plants, we first conducted an anatomic analysis of roots between susceptible and resistant *Vernicia* species and found that the pathogen *Fof*-1 was hindered in the root xylem of resistant *V. montana*. Based on our previous comparative transcriptomes^7^, we revealed that several candidate hub genes might be involved in resistance to *Fof*-1 infection. These hub genes included *D6 PROTEIN KINASE LIKE 2* (*D6PKL2*), *LRR-RLK2 CLAVATA2*, *diacylglycerol kinase* (*DGK*), *ETHYLENE RESPONSE FACTOR72* (*ERF72*) and *glycosyltransferase* (*GT*). Among these hub genes, *CLAVATA2*, *DGK*, *ERF72* and *GT1* have all been reportedly involved in pathogen resistance^11–14^. Specifically, it is well known that *D6PKL2* is involved in lateral root formation and root epidermal planar polarity^15,16^. However, whether *D6PKL2* is involved in disease resistance is poorly understood. Here, we revealed that *D6PKL2* was specifically expressed in root xylem and induced in response to *Fof*-1 infection in resistant *V. montana*. More importantly, the resistance ability of *VmD6PKL2* to *Fof*-1 in *Arabidopsis* and tomatoes was further verified. The study illuminates crucial information contributing to the control of *Fusarium* wilt disease in plants.

## Results

### *Fof*-1 failed to infect the lateral root xylem in the resistant *V. montana* trees

To track the infection process, the *Fof*-1 pathogen was transformed with the GFP (Green fluorescent protein) label. All positive transformants showed the expression of *GFP* and *hph* (Fig. S1a, b). In addition, the mycelium and conidium exhibited high fluorescence expression (Fig. S1c, d). After five subcultures, transformants named 3-1 and 3-10 showed stable *GFP* expression, similar phenotypes, and pathogenicity to the wild-type strain (Fig. S1e). Transformant 3-1 was selected for further infection experiments.

**Fig. 1:**
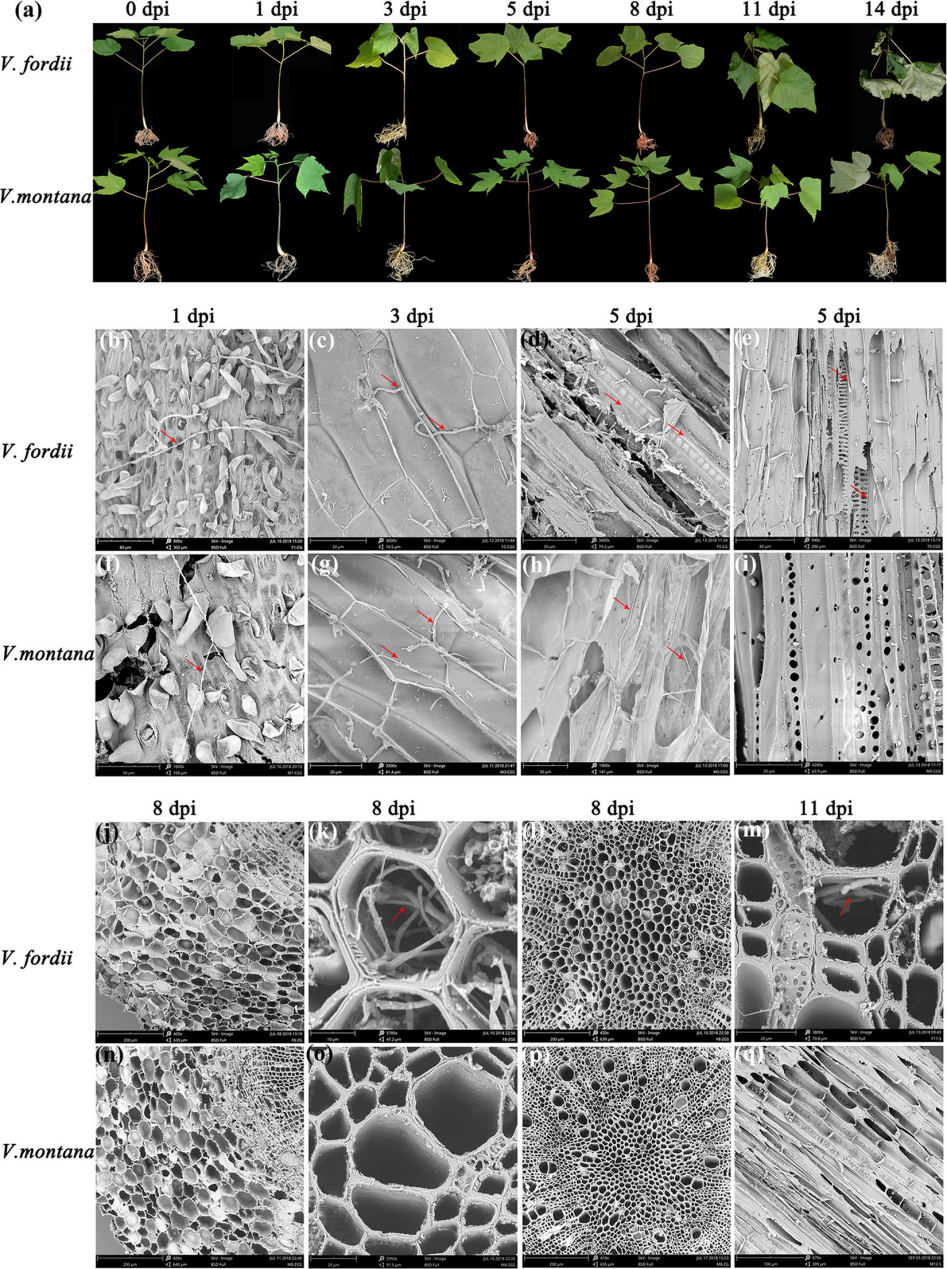
Histological analysis of *V. fordii* and *V. montana* after *Fof*-1 infection. **a** Phenotypic changes in *V. fordii* and *V. montana* infected with the wilt pathogen *Fof*-1 at different stages. **b**–**e**, **j**–**m** The development and infection process of *Fof*-1 in *V. fordii* at 1, 3, 5, 8, and 11 dpi. The tissues indicate epidermis (**b**), cortex (**c**), phloem (**d**), xylem (**e**) of the lateral root; phloem (**j**), xylem (**k**) and pith (**l**) of the main root; and xylem of the stem (**m**). **f**–**i**, **n**–**q**) The development and infection process of *Fof*-1 in *V. montana* at 1, 3, 5, 8 and 11 dpi. The tissues indicate epidermis (**f**), cortex (**g**), phloem (**h**), xylem (**i**) of the lateral root; phloem (**n**), xylem (**o**) and pith (**p**) of the main root; and xylem of the stem (**q**). The red arrows indicate hyphae of *Fof*-1

To explore the effective position or component preventing pathogen infection in resistant *V. montana*, the detailed infection process of *Fof*-1 in *V. fordii* and *V. montana* was comparatively observed (Fig. 1). Within 24 h after infection with *Fof*-1, spore germination and mycelium colonization were observed on the epidermis in both susceptible and resistant species (Fig. 1b, f; Fig. S2a, e). The hyphae initially attached themselves to the lateral root surface and grew along the junctions of epidermal cells to form a dense network intermingled with root hairs. Following surface colonization, direct penetration occurred primarily at the root hairs and their emergence sites of lateral roots (Fig. S2b, f). Hyphae became swollen, and appressorium-like structures were observed (Fig. S2c, f). At 3 dpi (days post-infection), the fungi propagated either intracellularly or intercellularly in the cortex of lateral roots in both *V. fordii* and *V. montana* (Fig. 1c, g). At 5 dpi, *Fof*-1 continued to grow centripetally and invaded the phloem and xylem of lateral roots in susceptible *V. fordii* (Fig. 1d, e), while the hyphae extended only to the phloem in resistant *V. montana* (Fig. 1h, i). At 8 dpi, *Fof*-1 spread longitudinally to the main roots through the xylem of the lateral root in susceptible *V. fordii*. The hyphae were confined to the xylem vessels of the main root (Fig. 1k), and no fungal proliferation was observed in the phloem or pith in *V. fordii* (Fig. 1j, l). In contrast, *Fof*-1 failed to invade the xylem of the lateral roots horizontally (Fig. 1°), and the pathogen could not move upwards through the phloem or pith in resistant *V. montana* (Fig. 1n, p). At 11 dpi, the fungi had spread to the stem xylem and caused wilting symptoms in susceptible *V. fordii* (Fig. 1a, m), while *Fof*-1 could still not infect the root xylem in resistant *V. montana* (Fig. 1q).

### *VmD6PKL2* transcript and protein specifically expressed in the root xylem of resistant *V. montana*

The full-length sequences of *VfD6PKL2* and *VmD6PKL2* were amplified from *V. fordii* and *V. montana*, respectively. Both the *VfD6PKL2* and *VmD6PKL2* genes contained 1833 bp of coding sequence (CDS) and encoded a 610-amino-acid protein with a predicted molecular mass of 67.1 kDa (Fig. S3a). The *D6PKL2* gene was a member of the PKC-like superfamily, with good local hydrophilicity but a lack of signal peptides and transmembrane helices (Fig. S3c–f). The active regions of proteins were concentrated mainly in 214-572 aa. Residue 442 within the conserved domain and seven other amino acids outside the conserved region were different in the two species (Fig. S3a, c). In terms of the gene structure, *D6PKL2* genes contained two introns in both *V. fordii* and *V. montana* (Fig. S3b). One intron lay in the 5′ untranslated regions (UTRs), and the other lay in the coding region. Sequencing of root tissues at four infection stages (0, 1, 8, 14 dpi) revealed no alternative splicing of *VfD6PKL2* or *VmD6PKL2* in response to *Fof*-1 infection (Fig. S3g).

**Fig. 2:**
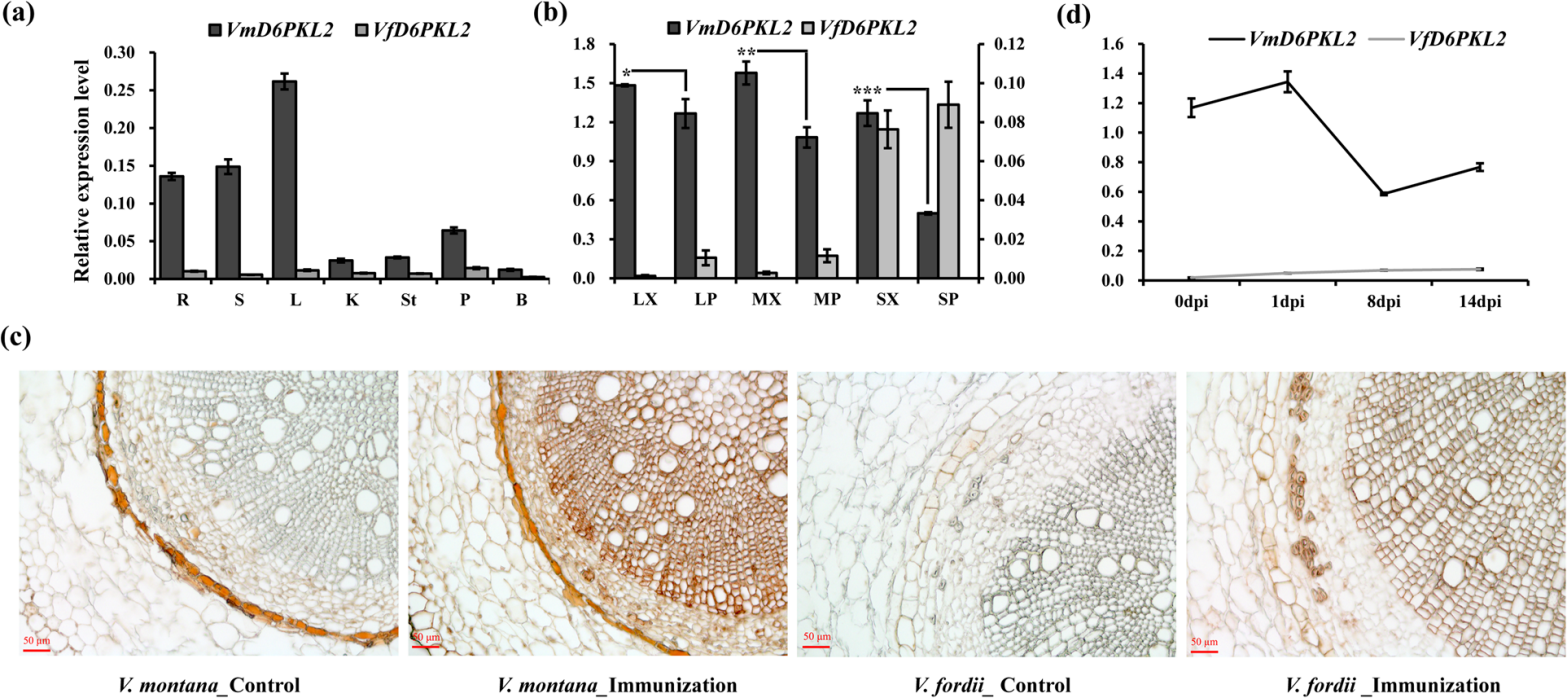
Expression pattern and immunolocalization analysis of *VfD6PKL2* and *VmD6PKL2*. **a** Tissue-specific expression patterns of *VfD6PKL2* and *VmD6PKL2* in root (R), stem (S), leaf (L), kernel (K), stamen (St), petal (P) and bud tissues (B). The *y*-axis indicates the relative expression level analyzed using qRT-PCR in triplicate. **b** Tissue-specific expression patterns of *VfD6PKL2* and *VmD6PKL2* in xylem of lateral root (LX), phloem of lateral root (LP), xylem of main root (MX), phloem of main root (MP), xylem of stem (SX), and phloem of stem (SP). The primary and secondary *y*-axes indicate the relative expression levels of *VmD6PKL2* and *VfD6PKL2*, respectively. Significant differences were determined by ANOVA and are represented with asterisks (*P < 0.05, **P < 0.01 and ***P < 0.001). **c** Immunolocalization analysis of *D6PKL2* in the main root of *V. montana* and *V. fordii*. Bars, 50 μm. **d** Expression trends of *VfD6PKL2* and *VmD6PKL2* in response to *Fof-1* at four infection stages

The tissue-specific expression patterns between the orthologous *VfD6PKL2* and *VmD6PKL2* were compared in seven tissues, including root, stem, leaf, kernel, stamen, petal and bud tissues (Fig. 2a). In general, the transcript abundance of *VmD6PKL2* was higher than the transcription abundance of *VfD6PKL2* in the seven tissues. The gene expression of *VmD6PKL2* in vascular tissues, such as roots, stems and leaves, was much higher than the gene expression of *VmD6PKL2* in nonvascular tissues. On this basis, we further investigated the expression levels of *VfD6PKL2* and *VmD6PKL2* in the xylem and phloem of lateral root, main root and stem tissues (Fig. 2b). The transcript abundance of *VmD6PKL2* in xylem was significantly higher than the transcript abundance in phloem in all of the vascular tissues examined. In contrast, the expression of *VfD6PKL2* in xylem was significantly lower than the expression of *VfD6PKL2* in phloem (*P* < 0.05). To detect the expression of D6PKL2 at the protein level, a qualified antiD6PKL2 antibody was prepared by which VmD6PKL2 can be specifically detected from root total protein (Fig. S4a, b). Immunolocalization analysis further revealed that VmD6PKL2 exhibited specifically higher expression levels in xylem in resistant *V. montana* at the protein level (Fig. 2c).

### *VmD6PKL2* as a novel resistance gene to *Fof*-1 in the root xylem of *V. montana*

To further explore the expression mode between *VfD6PKL2* and *VmD6PKL2* in response to the *Fof*-1 pathogen, expression patterns in root tissues at 0, 1, 8, and 14 dpi were investigated (Fig. 2d). We revealed that *VfD6PKL2* and *VmD6PKL2* displayed different expression patterns in response to *Fof*-1 infection. *VfD6PKL2* maintained a steady and significantly lower expression level than *VmD6PKL2* during pathogen infection. *VmD6PKL2* exhibited an increased expression pattern at the first infection stages at the transcript level. Western blot analysis further revealed that VmD6PKL2 was induced in response to *Fof*-1 infection (Fig. S4c).

**Fig. 3:**
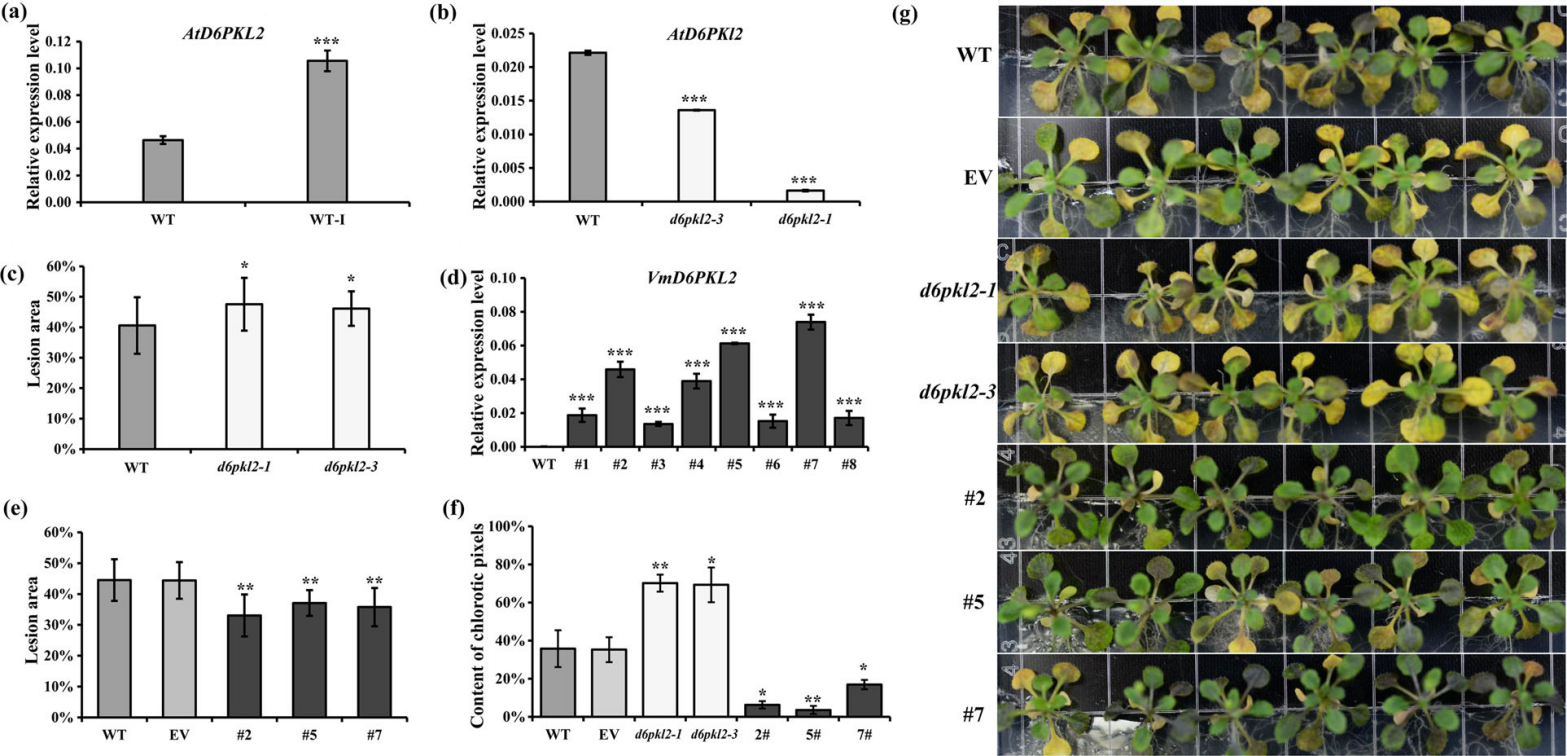
Resistance ability analysis of mutant and transgenic *Arabidopsis* overexpressing *VmD6PKL2* in response to *Fof*-1. **a** Changes in the relative expression levels of *AtD6PKL2* in wild-type *Arabidopsis* after *Fof*-1 infection. WT-I indicates plants infected with *Fof*-1 at 7 dpi. Relative expression levels of *AtD6PKL2* (**b**) and diseased leaf area at 7 dpi (**c**) of wild-type and homozygous mutants. The lesion area indicates the disease severity and was calculated based on the ratio of leaves showing disease symptoms using the open-source software ImageJ^55^. **d** Relative expression levels of *VmD6PKL2* in wild-type and eight transgenic lines (*35S:VmD6PKL2-GFP*). **e** Diseased leaf area of control plants (wild-type and empty vector) and the three selected transgenic lines at 7 dpi. **f** The content of chlorotic pixels of wild-type (WT), empty vector (EV), mutant lines (*d6pkl2-1*, *d6pkl2-3*), and *VmD6PKL2*-overexpressing transgenic lines (#2, #5, #7) infected with *Fof*-1 at 7 dpi. The analysis was conducted with reference to Pavicic et al.^56^. **g** Disease symptoms of mutant lines (*d6pkl2-1*, *d6pkl2-3*) and WT, EV, and *VmD6PKL2*-overexpressing transgenic lines (#2, #5, #7) infected with *Fof*-1 at 7 dpi. Three independent infection experiments were carried out with at least 24 plants per genotype in each experiment. Error bars represent ± SD. Significant differences were determined by ANOVA and are represented with asterisks (*P < 0.05, **P < 0.01 and ***P < 0.001)

*Arabidopsis* was used to identify whether *D6PKL2* was involved in the defense against *Fof*-1. First, the transcript abundance of *D6PKL2* in wild-type before and after infection was detected, and the results revealed that infection with *Fof*-1 significantly induced the expression of *D6PKL2* (Fig. 3a). Then, the homozygous null mutant lines of *d6pkl2*-1 and *d6pkl2-3* (Figs. S5a, 3b) as well as the wild-type were used to examine whether *d6pkl2* mutants displayed enhanced susceptibility to fungal pathogens. After infection with *Fof*-1 for approximately seven days, the *d6pkl2* mutant plants showed more severe yellowing symptoms and displayed significantly higher diseased leaf area than the wild-type plants (Fig. 3c, f).

To verify that *VmD6PKL2* played an essential role in resistance to *Fof*-1, the coding sequence of *VmD6PKL2* was overexpressed in wild-type *Arabidopsis* under the control of the CaMV35S promoter. *Arabidopsis* containing the empty vector was used as the control. The transgenic lines with obvious phenotypic defects due to ectopic expression were removed. Compared with the wild-type, eight T3 transgenic lines displayed significantly higher transcript abundance of *VmD6PKL2*. Lines #2, #5, and #7 with the highest expression levels were chosen for further study (Fig. 3d). In all three positive transgenic lines, green fluorescence was well expressed in the root tips (Fig. S5b). The lateral roots of the transgenic lines formed early and were better developed, and the growth rate was slightly faster than the growth rate of the wild-type plants. Upon pathogen infection, leaf chlorosis, yellowing and necrosis were evident in the control plants (wild-type and empty vector), whereas the *VmD6PKL2* transgenic lines exhibited a significantly reduced percentage of diseased leaf area and much milder symptoms (Fig. 3e, f). Taken together, these results imply that transgenic *Arabidopsis* overexpression of *VmD6PKL2* displayed enhanced resistance against *Fof*-1 infection.

**Fig. 4:**
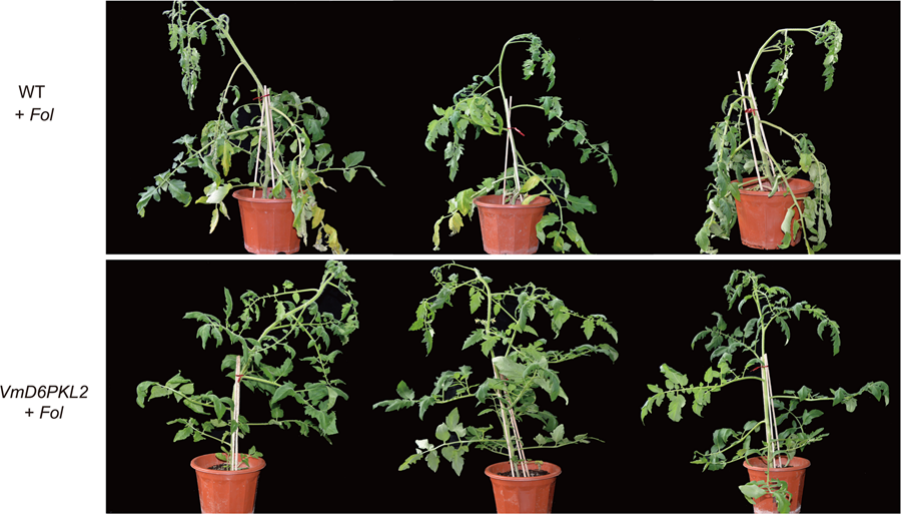
Resistance ability analysis of transgenic tomato lines overexpressing *VmD6PKL2* in response to pathogen *Fol*. WT + *Fol* represents wild-type tomato plants infected with pathogen *Fol*. *VmD6PKL2* *+* *Fol* indicates that transgenic tomato lines overexpressing *VmD6PKL2* were infected with *Fol*. The symptoms of plants after infection with *Fol* were observed at 17 dpi

To further verify the resistance ability of *VmD6PKL2*, we performed genetic transformation of *VmD6PKL2* in Ailsa Craig tomato plants. Positive transgenic tomato plants were identified using specific primers for *VmD6PKL2* (Fig. S6). Then, the transgenic tomatoes were further inoculated with *F. oxysporum* f. sp. *Lycopersici* (*Fol*). The *Fol* infection experiment results showed that the wild-type tomatoes infected with *Fol* had yellow leaves and wilting disease symptoms at 17 dpi, while the transgenic tomato lines grew well, and no wilt symptoms were observed after *Fol* infection (Fig. 4). These results indicated that the resistance ability was significantly increased in the transgenic tomatoes expressing *VmD6PKL2* compared with the wild-type tomatoes.

### VmD6PKL2 interacts with VmSYT3

**Fig. 5:**
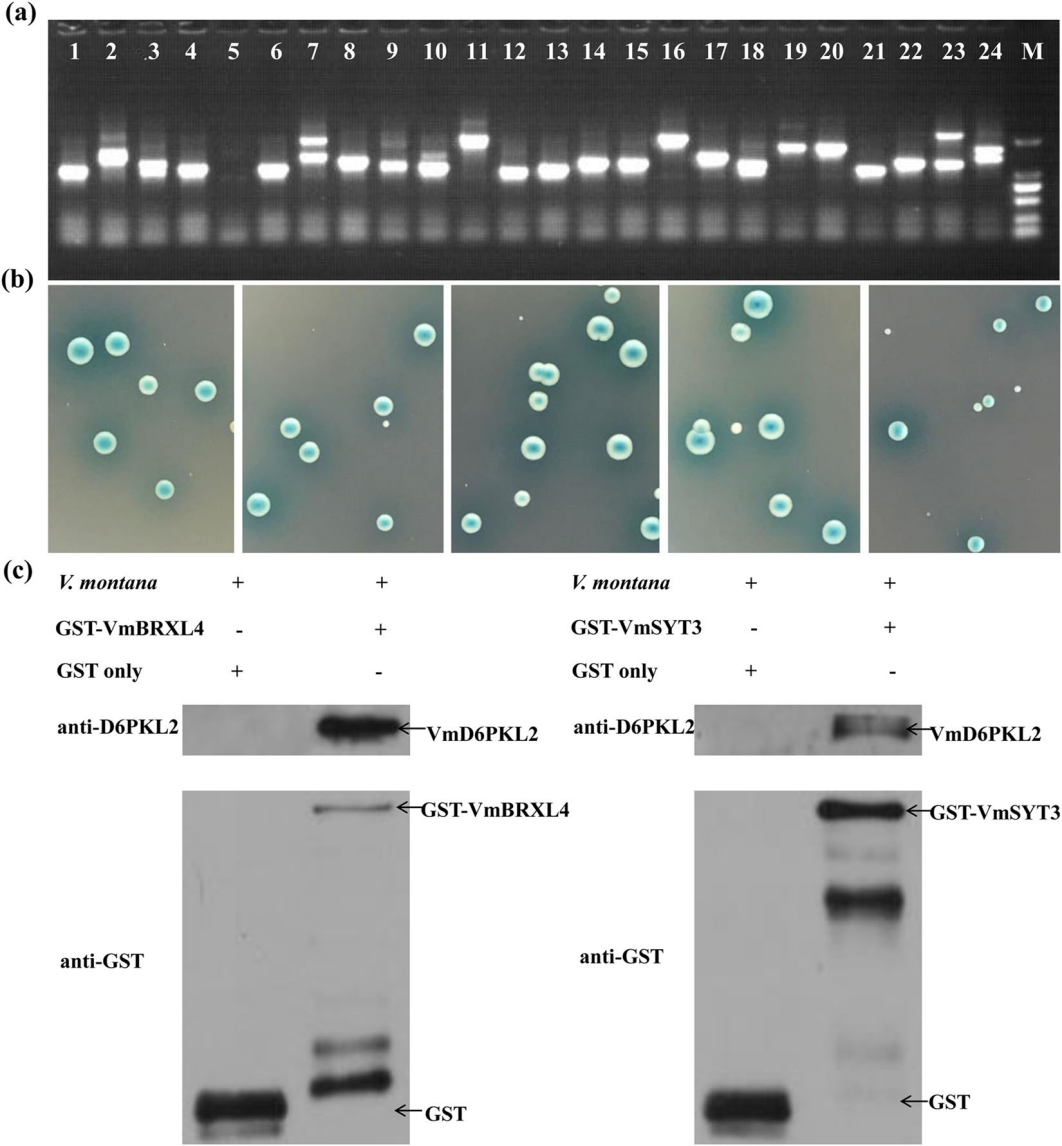
Yeast two-hybrid analysis of VmD6PKL2. **a** Calculation of recombination frequency and length of inserted fragments of yeast cDNA library. Lanes 1–24 represent the 24 randomly selected colonies, and M represents the DL2000 DNA marker. **b** The five nonredundant positive interaction proteins identified by one-to-one-interaction screening and sequencing analysis. From left to right: VmBRXL4, VmSYT3, VmABCI15, VmG6PD6, and VmHLB1. **c** Detection of the direct interaction between VmD6PKL2 and VmBRXL4 (left) or VmSYT3 (right). Plus and minus symbols indicate added and omitted components, respectively. The proteins detected by the antiGST or antiD6PKL2 antibody are indicated with arrows

To explore the interacting protein with VmD6PKL2, a yeast two-hybrid screen based on the infected root tissues in *V. montana* was performed. The 100% recombinant frequency and CFU greater than 7.6× 10^6^ revealed that the uncut and second cDNA libraries were constructed successfully (Fig. S7a, b). The further constructed yeast library exhibited 3×10^7^ cells/m library titer and 95% recombinant frequency, which met the requirements of library construction (Fig. 5a). The self-activation ability analysis suggested that VmD6PKL2 was transformed into the yeast strain successfully but could not activate the reporter gene by itself (Fig. S7c).

For bait-and-prey yeast-mating analysis, 32 colonies grown on triple synthetic dropout nutrient screening medium with blue color were identified as components that potentially interacted with VmD6PKL2. In addition, 20 out of the 32 colonies grew and turned blue on the quadruple SD nutrient screening medium (Fig. S7d). Finally, one-to-one cotransformation screening and sequencing analysis verified that five nonredundant candidate proteins, synaptotagmins 3 (SYT3), BREVIS RADIX-LIKE 4 (BRXL4), glucose-6-phosphate dehydrogenase 6 (G6PD6), ATP-binding cassette I15 (ABCI15), and hypersensitive to latrunculin B1 (HLB1), exhibited potential interactions with VmD6PKL2 in *V. montana* (Fig. 5b). The annotation, classification, and potential functions of these five genes are displayed in Table 1.

**Table 1:** Annotation and function of proteins interacting with VmD6PKL2

Name	Family	Function
SYT3	Calcium-dependent lipid-binding (CaLB domain) family protein	A paralog of SYT1 is involved in fungi defense^37^; the putative calcium sensor catalyze Ca^2+^ -triggered vesicles fusion^51^.
BRXL4	BRX gene superfamily	A paralog of BRX acts as a molecular rheostat to modulate auxin efflux dynamically^52^; interact with the pathogens defense gene RLM3^53^.
G6PD6	G6PD-C superfamily	.Positive resistance gene to bacterial pathogen and root-knot nematode^42,43^
ABCI15	ABC transporter family	Lipidic metabolic intermediates transport; some ABC transporters involved in pathogen resistance^46^.
HLB1	Tetratricopeptide repeat (TPR)-like superfamily	Modulate the trans-Golgi network/early endosome (TGN/EE)^54^.

### VmD6PKL2 directly captures VmSYT3 in vitro

The direct interactions between VmD6PKL2 and VmSYT3 or VmBRXL4 were confirmed by GST pull-down assays in vitro. First, the ORF sequences of the *VmSYT3* gene encoding a 538-amino-acid protein and the *VmBRXL4* gene encoding a 377-amino-acid protein were amplified to construct recombinant plasmids PGEX-GST-VmSYT3 and PGEX-GST-VmBRXL4. The prediction of transmembrane helices revealed that the seventh to 29^th^ amino acids of VmSYT3 made up the transmembrane region (Fig. S7e). We therefore truncated the amino acids and selected the region of 50 aa to 538 aa to fuse with GST and express recombinant protein (Fig. S8a). SDS-PAGE and immunoblotting with antiGST antibody showed that GST tags could be detected in the purified GST-VmSYT3 and GST-VmBRXL4 proteins as well as GST protein (Fig. S8b, c). The molecular mass of GST-VmBRXL4 was approximately 70 kDa, and GST-VmSYT3 was approximately 81 kDa, which was consistent with the target protein (Fig. S8c).

GST-VmSYT3 or GST-VmBRXL4 fusion protein bound to glutathione-coated beads was further assayed for the ability to pull down VmD6PKL2. As displayed in Fig. 5c, GST-VmSYT3 protein and GST protein were detected by antiGST antibody, suggesting that these two proteins bound to Sepharose beads successfully. Immunoblot analyses using an antiD6PKL2 antibody also demonstrated that VmD6PKL2 was pulled down by GST-VmSYT3 but not by GST. Similarly, Western blot assay detected the existence of GST and GST-VmBRXL4 and verified that VmD6PKL2 could be pulled down only by GST-VmBRXL4 in Fig. 5c, indicating that GST-VmBRXL4 binds to VmD6PKL2 in vitro. All these results revealed that VmD6PKL2 can directly interact with VmSYT3 or VmBRXL4.

### *VmD6PKL2* suppresses *VmSYT3*-mediated negative regulation

**Fig. 6:**
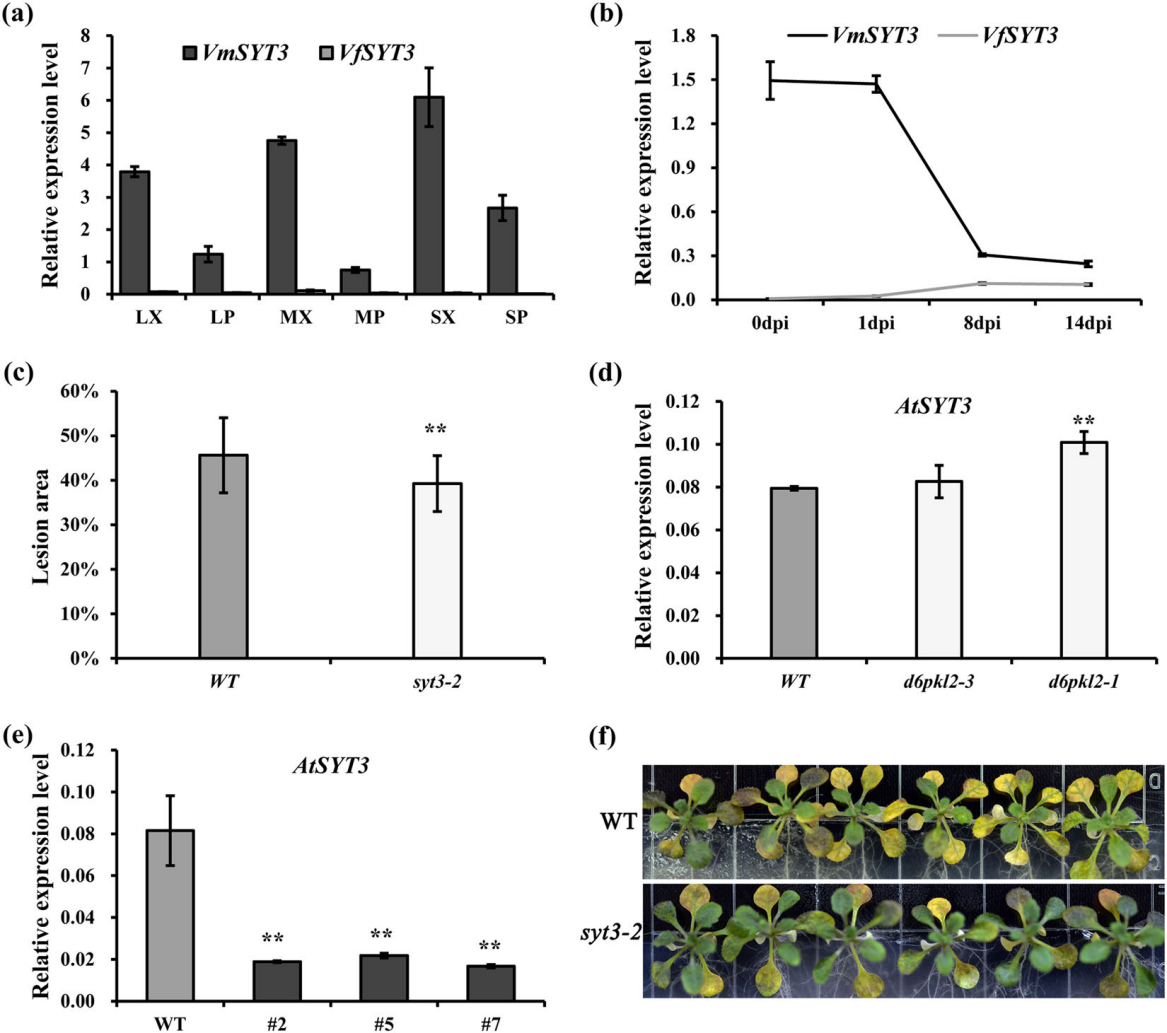
Resistance ability analysis of *syt3* mutants in response to *Fof*-1 and the regulation of *SYT3* by *D6PKL2*. Three independent infection experiments were performed. Significant differences are indicated with asterisks. **a** Tissue-specific expression patterns of *VfSYT3* and *VmSYT3* in xylem and phloem of vascular tissues. LX, xylem of lateral root; LP, phloem of lateral root; MX, xylem of main root; MP, phloem of main root; SX, xylem of stem; SP, phloem of stem. **b** Expression trends of *VfSYT3* and *VmSYT3* in response to *Fof*-1 at four infection stages. Diseased leaf area (**c**) and disease symptoms (**f**) of the *syt3-2* mutant and wild-type infected with *Fof*-1 at 7 dpi. Changes in the relative expression levels of *AtSYT3* in *d6pkl2* mutants (**d**) or transgenic *Arabidopsis* overexpressing *VmD6PKL2* (**e**) compared with wild-type *Arabidopsis*. Significant differences were determined by ANOVA and are represented with asterisks (**P < 0.01)

To identify whether *SYT3* was involved in the defense against *Fof*-1, tissue-specific expression patterns of both *VfSYT3* and *VmSYT3* in vascular tissues were investigated. Similar to the expression modes of *Vf/VmD6PKL2*, *VmSYT3* was specifically expressed in the xylem, while *VfSYT3* showed almost no expression in any of the vascular tissues (Fig. 6a). In addition, after infection with *Fof*-1, the transcript abundance of *VmSYT3* was gradually downregulated, whereas little change was displayed in the expression of *VfSYT3* (Fig. 6b). This result suggested that the root xylem-specifically expressed *VmSYT3* may act as a negative regulator of wilt disease.

Wild-type *Arabidopsis* and *syt3* mutants were used to detect the resistance ability against *Fof*-1. Homozygosity verification showed that all of the *syt3-2* and several *syt3-3* mutant lines were homozygous, but all *syt3-1* were heterozygous (Fig. S9a, b). In addition, qRT-PCR analysis revealed that *syt3-2* but not *syt3-3* displayed significantly lower expression levels than the wild-type (Fig. S9c). Therefore, homozygous *syt3-2* mutants were selected for further investigation. Inoculation results indicated that the *syt3-2* mutant lines showed significantly lower diseased leaf area and lighter wilting disease symptoms than the wild-type plants (Fig. 6c, f), suggesting that *SYT3* negatively regulates defense responses.

To examine the regulation of *SYT3 by VmD6PKL2*, we detected the transcript abundance of *AtSYT3* in *d6pkl2* mutants and transgenic *Arabidopsis* overexpressing *VmD6PKL2*. The results revealed that the mutation of *AtD6PKL2* increased the expression level of *AtSYT3* (Fig. 6d), and, in contrast, *AtSYT3* was significantly downregulated in all three transgenic *Arabidopsis* lines overexpressing *VmD6PKL2* (Fig. 6e).

## Discussion

Although numerous studies on plant *Fusarium* wilt disease have been performed^5,17^, the detailed process of root infection by the pathogenic *F*. *oxysporum* and the genetic basis for resistance or susceptibility of plants to wilt diseases remain obscure. In this study, the divergent infection process of *Fof*-1 into susceptible *V. fordii* and resistant *V. montana* were presented, respectively (Fig. 1). This is the first known report detailing the systemic infection process of *F. oxysporum* within trees. Infections took place through the secondary roots but not the main root, which was consistent with the finding by Trujillo, Snyder^18^. Subsequently, after penetrating the epidermis of lateral roots, *Fof*-1 grew centripetally and infected the cortex, phloem and xylem in *V. fordii* (Fig. 1). Colonization in xylem vessels is a crucial step of the infection process for the vascular wilt disease pathogen. From this stage on, the fungi were confined to the xylem vessels and moved upwards to the main root. Sporulation and germination of secondary mycelium are considered crucial for rapid upward colonization^9^. The microconidia, carried upwards by the xylem stream, were observed prior to mycelium infection in the xylem of the stem (Fig. 1). In *V. fordii*, chlamydospores were produced on the lateral or terminal of the hyphae as they invaded the xylem of the lateral root (Fig. S2d, h). Interestingly, chlamydospores were generated only when the fungi enter the xylem in all early infection processes, contrary to the common assumption that they are produced only after the death of the plant^19^. The wilt disease development of branches and leaves was significantly correlated with the infection site of roots, consistent with the mode of pathogen expansion observed in the field. In detail, if the fungi only infected the lateral roots on one side of the tree, the main root in that direction was infected; accordingly, the vascular bundles of the trunk, branches and leaves in that position became necrotic and wilted. If half of the lateral roots and main root were infected, then half of the trunk, branches and leaves were withered from the whole tree.

The initial infection process of *Fof*-1 in resistant *V. montana* was similar to the initial infection process in susceptible *V. fordii*. In detail, the hyphae can penetrate the epidermis, invade the cortex, and enter the phloem of lateral roots. However, then, fungal propagation appeared to be halted. The hyphae could not spread sequentially to the xylem vessels of lateral roots and main root (Fig. 1). These results indicate that the resistance of *V. montana* to *Fusarium* wilt disease does not occur at the penetration stage but through inhibiting pathogen progression. In addition, the xylem of the lateral root of *V. montana*, exposed directly to the pathogen, showed almost no fungal infection (Fig. S2g). These results reveal that the resistance of *V. montana* to wilt disease is closely related to xylem, which probably inhibits the invasion of *Fof*-1.

Inoculation experiments showed that *V. montana* was resistant to *Fusarium* wilt disease not only under natural conditions but also under growth-chamber conditions. Correspondingly, *V. fordii* is highly susceptible to *Fof*-1 under natural and controllable conditions, revealing that genetic factors affect the resistance or susceptibility of plants to fungi to a great extent. Our previous study suggested that *VmD6PKL2* may be a hub gene in resistance to *Fof*-1 in *V. montana*^7^. D6PKL2, together with D6PK, D6PKL1, and D6PKL3, forms the D6PK subfamily within the AGCVIIIa family of *Arabidopsis* serine/threonine protein kinases^20^. AGC kinases are named after protein kinase A (PKA), cyclic GMP-dependent protein kinases (PKG) and protein kinase C (PKC)^21^. Many AGC kinases, such as protein kinase 1 (*OsPdk1*), oxidative signal inducible 1 (*OXI1*), and *TaAGC1*, have been suggested to defend against pathogens and plant immunity^22–25^. Studies on *D6PKL2* and the related D6PKs have focused on the control of auxin transport-dependent growth^26^. Auxin plays important roles in the differentiation of xylem cells, radial pattern formation of vascular bundles and molding plant-pathogen interactions^27–29^. *d6pk012* triple mutants are severely impaired in several developmental processes, including tropic responses, lateral root formation and phototropic hypocotyl bending^16,30^. However, to date, it is unknown whether *D6PKL2* is involved in disease resistance. The data here indicated that *VmD6PKL2*, rather than *VfD6PKL2*, was induced in response to fungal infection, and *VmD6PKL2* transcripts and protein were both specifically expressed in the root xylem (Fig. 2). Furthermore, transgenic *Arabidopsis* and tomatoes overexpressing *VmD6PKL2* exhibited high resistance ability, while the mutant lines were more susceptible to the pathogen (Fig. 3). Interestingly, *Arabidopsis atd6pkl2* and overexpression lines exhibited subtle disease phenotypes compared with the overexpression of *VmD6PKL2* in tomatoes. Based on our anatomic analysis (Fig. 1), the xylem of the lateral roots is the key position for the resistance ability to *Fof*-1 in *V. montana* trees. We therefore suspected that the relatively weak secondary xylem of *Arabidopsis*, not as strong as the secondary xylem of tung trees and tomatoes, may be the reason for the subtle disease phenotypes.

**Fig. 7:**
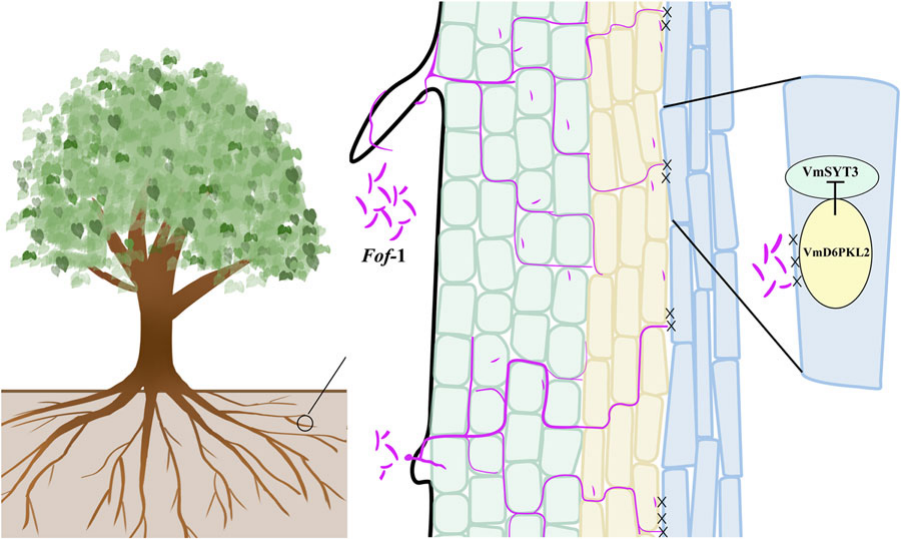
A proposed model for the infection process of pathogen *Fof*-1 and potential defense response of *V. montana*. The microconidia of *Fof*-1 germinated and colonized the epidermis of the lateral root. After penetration into root hairs, hyphae continue to grow centripetally and infect the cortex and phloem, but further invasion into xylem is blocked. VmD6PKL2 positively regulates resistance against *Fusarium* wilt disease through suppression of *VmSYT3*-mediated negative regulation

How is *VmD6PKL2* positively involved in lateral root xylem resistance to the pathogen? SYT3, one of five synaptotagmins (SYTs) encoded by *Arabidopsis*^31^, was identified to directly interact with VmD6PKL2 in resistant *V. montana* (Fig. 4b, c). Mammalian SYTs act as Ca^2+^ sensors to trigger synaptic vesicle fusion and hormone secretion^32^. In addition, plant SYTs containing two C-terminal calcium-binding domains (C2A and C2B), such as mammalian Syts, may also act as a calcium sensor^33^. Ca^2+^ sensors can sense cytosolic Ca^2 + ^elevations rapidly caused by a variety of stimuli^34^ and give rise to altered gene expression patterns and protein phosphorylation^35^. In addition, calcium signals are crucial in plant defense-signaling pathways^35^ and may trigger protein kinases^36^. These results led us to hypothesize that after *Fof*-1 infection, VmSYT3 in xylem recognized calcium influx and transmitted the signal to *VmD6PKL2* in a Ca^2+^ -dependent manner, which activated the expression of *VmD6PKL2* (Fig. 7). In our study, we further identified that *SYT3*, similar to the function of *AtSYT1*^37^, negatively defended against Fusarium wilt disease. In addition, overexpression of VmD6PKL2 significantly downregulated the expression level of *SYT3* (Fig. 6). These results suggested that *VmD6PKL2* activated by VmSYT3 could in turn suppress *At*SYT3-mediated negative regulation through posttranscriptional modification.

Phosphorylation is critically important for the regulation of protein function^38^. Many diseases occur as the result of mutated phosphorylation sites^39^. D6PKs are suggested to be key regulators of PIN-FORMED (PIN1) phosphorylation^40^. In our study, some of the other candidate proteins that interact with VmD6PKL2, including G6PD6 and ABCI15, have a known or suspected role in plant defense in a phosphorylation-dependent manner (Table 1). G6PD, a key enzyme of the oxidative pentose phosphate pathway, exhibits pathogen-inducible activity and provides equivalents important for defense responses^41^. Mutation of cytosolic isoform G6PD6 resulted in increased susceptibility of *Arabidopsis* to the *Pseudomonas syringae* and root-knot nematode^42,43^. Phosphorylation stimulated the activity of G6PD6, which is critical for plant adaptation to abiotic and biotic stresses^44^. ABC transporters regulate the overall development and stress tolerance of plants via the transport of cellular building blocks and secondary metabolites^45,46^. Many ABC transporters have been identified to confer resistance to multiple fungal pathogens and insects^47–49^. Phosphorylation is constitutive and required for full transporter activity^38^. These results lead us to speculate that, in addition to the suppression of *VmSYT3*-mediated negative regulation, *VmD6PKL2* could positively regulate disease resistance through the activation of downstream defense-related genes in a phosphorylation-dependent manner (Fig. 6).

In conclusion, the susceptible and resistant plant sister species make it possible for us to reveal that *Fof*-1 failed to invade into the lateral root xylem of resistant tree species, while it spread upwards through the root xylem in susceptible tree species. This finding reminds us that the lateral root xylem is a key barrier for *Fusarium* pathogen infection. More importantly, *VmD6PKL2*, specifically expressed in the root xylem and induced in response to *Fof*-1 infection, acts as a resistance gene against *Fof*-1 by suppressing *VmSYT3*-mediated negative regulation in the lateral root xylem. This result further illuminates our understanding of the basis of genetic resistance against *Fusarium* in the root xylem of resistant plant species. The findings provide novel insight into *Fusarium* wilt resistance in plants.

## Materials and methods

### Plant materials and growth conditions

Tung trees and *Arabidopsis* were used in this work. Seeds of *V. fordii* and *V. montana* were harvested in Guangxi Zhuang Autonomous Region, China. They were first immersed in water overnight, sterilized in 0.2% potassium permanganate for 1 h, and subsequently washed three times in sterile water. The surface-sterilized tung tree seeds were grown in sterile soil under growth-chamber conditions at 26 °C with a 16 h light/8 h dark photoperiod and 60% relative humidity. The *D6PKL2* (At5g47750) and *SYT3* (At5g04220) mutants, *d6pkl2-1* (SALK_011339C), *d6pkl2-2* (SALK_099935), *d6pkl2-3* (SALK_086127), *syt3-1* (SALK_005585C), *syt3-2* (SALK_124835C), and *syt3-3* (SALK_077067), were ordered from the *Arabidopsis* Biological Resource Center (ABRC).

The three-primer method was used to confirm the homozygous mutants. The primers were designed at the website http://signal.salk.edu/tdnaprimers.2.html. The T-DNA insertion site of the homozygous mutants *d6pkl2-1*, *d6pkl2-3*, and *syt3-2* used for infection investigation was exon. *Arabidopsis* wild-type (WT) Columbia (Col-0) and mutant seeds were surface-sterilized in 75% ethyl alcohol for 30 min and germinated on 1/2 Murashige and Skoog (MS) medium containing 1.5% sucrose and 0.5% agar under a 16 h light/8 h dark cycle and 60%-75% relative humidity at a temperature of 23 °C.

### *Agrobacterium tumefaciens*-mediated transformation of *Fof*-1

The *Fof-1* pathogen was isolated from infected tung tree plants in Guangxi Zhuang Autonomous Region, China^7^ and maintained on potato dextrose agar (PDA) at 28 °C. Conidial spores were collected with sterile distilled water and diluted to the desired concentration of 1 × 10^6^ spores per ml. The *A. tumefaciens* strain AGL-1 carrying the plasmid pKD1-GFP was used for the genetic transformation of *Fof*-1. Transformants were selected on PDA containing hygromycin B (100 μg ml^−1^). The expression of *GFP* and the hygromycin B phosphotransferase gene (*hph*) of transformants were further detected by PCR amplification. Meanwhile, the fluorescence expression of the mycelium and conidium of transformants was assessed by a Zeiss LSM700 confocal laser scanning microscope (Carl Zeiss Inc., Jena, Germany). GFP fluorescence was excited at 488 nm lasers and detected at 500–530 nm. After five subcultures on PDA plates without hygromycin B, transformants were reinoculated on PDA containing hygromycin B to detect their fluorescence stability. Eventually, the transformants that exhibited high expression of GFP and equal phenotype and pathogenicity with wild-type were selected for pathogen infection assays.

### Fungal infection assays of tung trees and histological analysis

Seedling plantlets of two-month-old *V. fordii* and *V. montana* with 4–5 leaves were used. The sterile roots of chosen plantlets were drilled with a needle and then placed into the conidial suspension (10^6^ spores per ml) for 30 min, while control plants were dipped in sterile distilled water. After inoculation, the plants were replanted in the growth room with 85% humidity.

The root tip, lateral root without secondary structure, main root with secondary structure, stem and leaf tissues at 0, 1-, 3-, 5-, 8-, 11-, and 14-days post-infection (dpi) were sampled to observe the different infection processes of *Fof*-1 in *V. fordii* and *V. montana*. Four inoculated plants and three uninfected plants were harvested at each sampling time. For confocal laser scanning microscopy (CLSM) analysis, samples were hand-sectioned into approximately 1-mm-thick slices. Confocal images were acquired on a Zeiss LSM700 confocal laser scanning microscope as described above. Endogenous plant autofluorescence was recorded from 530 to 690 nm.

For scanning electron microscopy (SEM) analysis, vacuumed sections were fixed in 2.5% glutaraldehyde overnight at 4 °C. Samples were rinsed with PBS three times and then dehydrated in a graded ethanol series (30, 50, 70, 80, 90, 95, and 100%) for 15 min per gradient. Afterwards, samples were freeze-dried using a CO_2_ critical point dryer (Quorum, London, UK) and coated with gold-palladium using a gold spraying instrument (Zhongke Keyi, Beijing, China). Finally, the characteristics of the sample tissues were observed, and electron microphotographs were obtained using a scanning electron microscope (Phenom-world, Eindhoven, Netherlands).

### RNA extraction and qRT-PCR analysis

A series of tissues from tung trees and *Arabidopsis* was harvested and ground in liquid nitrogen immediately. Among these tissues, root, stem, leaf, kernel, stamen, pistil and bud tissues, as well as xylem or phloem of main root, lateral root and stem in uninfected *V. fordii* and *V. montana* were used for tissue-specific expression analysis. The roots of control and inoculated tung trees at 1, 8, and 14 dpi were used for expression trend analysis. Total RNA extraction and quality detection and first-strand cDNA synthesis were performed according to Zhang (2016). qRT-PCR analysis was used to examine the relative expression levels^6^. *AtUBQ5* and *EF1a* genes from *Arabidopsis* and tung tree, respectively, were used as internal controls. Three independent biological replicates from each sample were used and analyzed in technical triplicates. The 2^-ΔΔCT^ calculation method was used to determine the relative expression levels. Primers were designed using Primer Premier 5.0 (Premier Biosoft, Palo Alto, CA, USA).

### Gene isolation and sequence analysis

The full-length ORF sequences of the *VfD6PKL2* and *VmD6PKL2* genes were amplified from cDNA templates of roots in *V. fordii* and *V. montana*, respectively. Purified sequences were cloned into the pMD18-T vector and transferred into *E. coli* DH5α. The inserted gene fragment of positive transformants was confirmed by sequencing and further BLASTX analysis on NCBI (http://www.ncbi.nlm.nih.gov/). In addition, the Conserved Domain Database (https://www.ncbi.nlm.nih.gov/cdd/), SignalP 4.1 Server (http://www.cbs.dtu.dk/services/SignalP/), TMHMM (http://www.cbs.dtu.dk/services/TMHMM/), and HMMTOP 2.0 (http://www.enzim.hu/hmmtop/html/submit.html) were used to analyze the encoded amino acid sequences. The sequences of *VfD6PKL2* and *VmD6PKL2* of tung trees infected with *Fof*-1 at 0, 1, 8, and 14 dpi were used for alternative splicing analysis. Genomic DNA of *V. fordii* and *V. montana* was extracted using a CTAB Plant DNA kit (Aidlab Biotech, Beijing, China). The intron fragments of *VfD6PKL2* and *VmD6PKL2* were amplified via PCR and then sequenced as described above.

### Antibody preparation, Western blotting and immunolocalization

An antiD6PKL2 polyclonal antibody was developed specifically against the C-terminal peptide antigen APDKKGSDNY (amino acids 595 to 604) of D6PKL2. In detail, the specific peptide sequence was synthesized and detected by HPLC. In addition, the peptide coupled with the immunoenhancer vector via cysteine was used to immunize rabbits. The specificity and titer of VmD6PKL2 antiserum were confirmed by ELISA. Total proteins were extracted from the root tissue of *V. montana* according to the instructions of YeastBuster™ Protein Extraction Reagent (Merck, Darmstadt, Germany). To validate the specificity of the antiD6PKL2 antibody, total proteins were stained by Coomassie blue after SDS-PAGE, and VmD6PKL2 was detected by antiD6PKL2 antibody in Western blotting analysis. β-Tubulin was used as internal control.

The lateral roots and main roots of *V. fordii* and *V. montana* were cut into 2–3 mm pieces and immediately fixed in FAA (50% ethyl alcohol, 10% formaldehyde, 10% glycerol, and 5% acetic acid) for at least 24 h. After gradual dehydration in gradient concentrations of tert-butanol and ethanol solutions, samples were kept in paraffin at 60 °C for 48 h. The embedded tissues were further sliced into 12-μm-thick sections. After dewaxing with xylene, the sections were rehydrated in gradient ethanol and 0.1 M PBS. The blocked slides were inoculated overnight in 1:500 diluted antiD6PKL2 antibodies at 4 °C and then inoculated in 1:50 diluted HRP-labeled goat antirabbit IgG (Beyotime Biotechnology, Shanghai, China) for 2 h. Finally, the rinsed slides were stained with a DAB horseradish peroxidase color development kit (Beyotime Biotechnology, Shanghai, China) for 15 min and observed under a Leica DM4000B light microscope (Leica, Wetzlar, Germany).

### Fungal infection assays of mutant and transgenic *Arabidopsis*

The coding sequence of *VmD6PKL2* (without the stop codon) was linked to the linear vector pCambia1300-GFP/C using Trelief™ SoSoo Cloning Kit (TSINGKE, Beijing, China). The successfully constructed vector was transformed into *Arabidopsis* wild-type Col-0 mediated by *A. tumefaciens* GV3101 using the floral dip method^50^. The harvested *Arabidopsis* seeds were screened on 1/2MS medium with 20 μg·ml^−1^ hygromycin B. The positive transgenic lines (*35* *S:VmD6PKL2-GFP*) were further confirmed by PCR analysis of *VmD6PKL2* and *GFP*. All the positive transformants were cultivated to the T3 generation, and the expression of *VmD6PKL2* in transgenic *Arabidopsis* was analyzed using qRT-PCR. Subsequently, three independent transgenic lines with higher *VmD6PKL2* expression levels were selected for fungal infection. Transgenic lines harboring the empty vector (*35* *S:GFP*) were also obtained and used as controls.

The seedlings of WT, transgenic lines (*35* *S:GFP* and *35* *S:VmD6PKL2-GFP*), *d6pkl2* and *syt3* mutants grown on 1/2 MS medium for 7 days were transferred to square dishes containing the same medium. After five days of growth, each *Arabidopsis* plant was inoculated with a piece of *F. oxysporum* f. sp. *fordiis* (*Fof*-1) with the diameter of 5 mm. Three independent experiments were performed with at least 24 plants per genotype in each experiment. Disease progression and chlorosis development were observed and recorded. Disease severity was assessed based on the ratio of yellow leaves to the number of total leaves per plant. Statistically significant differences among independent genotypes were estimated by one-way analysis of variance (ANOVA). Plants were sampled at 0 and 7 dpi for the measurement of the change in expression.

### Infection assays of transgenic tomatoes with *VmD6PKL2*

The CDS of *VmD6PKL2*-GFP was inserted into the pCAMBIA1300 vector under the control of the Camv35S promoter. The Ailsa Craig tomato variety was used for genetic transformation, and transformation was conducted using the *A. tumefaciens*-mediated leaf disc method. Transgenic tomato plants (*35S:VmD6PKL2-GFP*) were further screened using hygromycin B and finally identified using specific primers for hygromycin and *VmD6PKL2* (Table S1) using PCR. The transgenic and wild-type seedlings were grown on 1/2 MS medium for 7 days and then transferred to sterile soil for cultivation for approximately two weeks. The roots of wild-type and transgenic tomatoes were drilled with a needle and then injected with conidial suspension (10^6^ spores per ml) of *Fol* around the roots. After inoculation, the plants were cultivated under growth-chamber conditions at 26 °C with a 16 h light/8 h dark photoperiod and 60% relative humidity. The symptoms after infection were observed and recorded from 0 to 17 dpi.

### Construction of cDNA library for yeast two-hybrid assay

Root tissue of *V. montana* after *Fof*-1 infection was used for the extraction of total RNA. The mRNAs were isolated and purified from qualified RNA according to the protocol of the Oligotex mRNA Midi Kit (Qiagen, Hilden, Germany). An uncut-type cDNA library was constructed using the CloneMiner II cDNA Library Construction Kit (Invitrogen, Carlsbad, CA, USA). The plasmids of the qualified uncut cDNA library were extracted for recombination with the yeast expression vector pGADT7-DEST. The recombinant vector was transformed into competent *E. coli* DH10B cells by electroporation. Ten microliters of the transformed cell culture was diluted 1:1000, and 50 µl of the solution was spread on LB medium with kanamycin. After incubating overnight at 37 °C, colony-forming units (CFU) of the second cDNA library were calculated as follows: colonies/50 µl medium × 1000 times ×10^3^ µl × total volume of the library (ml). Twenty-four randomly selected colonies were examined for calculation of recombination frequency and length of inserted fragments by colony PCR amplification. The extracted plasmids of the second cDNA library were subsequently transformed into the competent cells of yeast Y187 with the PEG-LiAc method. After the selection of positive transformants with SD/-Leu plate medium, the CFU and recombination frequency of the yeast library were calculated.

### Yeast two-hybrid screening

The coding sequence of *VmD6PKL2* linking *Eco*R I and *Bam*H I restriction sites was inserted into the bait-vector PGBKT7. To detect the self-activation ability of VmD6PKL2, PGBKT7-VmD6PKL2 and the positive-control combination of PGBKT7-53 and PGADT7-T, as well as the negative-control combination of PGBKT7-Lam and PGADT7-T, were individually transfected into the competent cells of yeast strain Y2H GOLD. SD/-Trp/X-α-Gal and SD/-Trp/-Leu/X-α-Gal medium was used to screen the bait transformants PGBKT7-VmD6PKL2 and the control transformants. In addition, the bait yeast strain Y2HGold containing PGBKT7-VmD6PKL2 and library yeast strain Y187 containing PGADT7-cDNA were mated and screened by SD/-Trp-Leu-His/X-α-Gal/AbA medium to identify the components that potentially interact with VmD6PKL2. The positive colonies were further cultured on SD/-Ade-Trp-Leu-His/X-α-Gal/AbA medium, and the expression of the reporter gene LacZ was confirmed according to the blue color of the colony. Prey plasmids PGADT7-cDNA were extracted from positive blue colonies, which were further retransfected into Y2HGold possessing bait plasmid PGBKT7-VmD6PKL2 for one-to-one-interaction screening. Inserted cDNA sequences in prey plasmids of positive colonies were sequenced and analyzed with the BLASTX program available at NCBI.

### GST pull-down assay

The ORF sequences of *VmBRXL4* and *VmSYT3* were amplified from cDNA templates. The encoded amino acid sequences of these two genes were analyzed in the same way as *D6PKL2*. Then, the appropriate fragment of these two genes linking *Eco*R I and *Xho*I restriction sites was cloned into the prokaryotic expression vector PGEX-6P-1. Successfully constructed recombinant plasmids PGEX-GST-VmSYT3 and PGEX-GST-VmBRXL4 were transformed into *E. coli* Rosetta for protein expression. IPTG was used to induce the high expression of GST-VmBRXL4 and GST-VmSYT3. The inclusion body proteins were purified, renatured, and finally analyzed using SDS-PAGE and western blotting assays.

Fifty nanograms of GST protein, as a control, and 30 µl of strains expressing GST-VmSYT3 or GST-VmBRXL4, as test groups, were incubated with glutathione resin-bound proteins for 1 h at 4 °C. After incubation, the unbound proteins were removed by TBS buffer (10 mM Tris, 150 mM NaCl, pH 8.0) five times. Then, 50 µg of total protein from the root tissue of *V. montana* was incubated with the control and test groups separately overnight at 4 °C. The beads were then washed three times with TBS buffer and lysed by RIPA buffer (50 mM Tris-HCl, pH 8.0, 150 mM NaCl, 2 mM EDTA, 1% NP-40, 0.5% sodium deoxycholate, 0.1% SDS) and 2× loading buffer (100 mM Tris-HCl, 200 mM DTT, 4% SDS, 0.2% bromophenol blue, 20% glycerol). After boiling for 10 min and centrifugation of the lysate, the supernatant was collected. Finally, the pulled-down complexes were subjected to SDS-PAGE and analyzed by Western blot using antiGST (1: 50,000) and antiD6PKL2 (1: 1,000) antibodies.

### Accession numbers

These sequence data have been submitted to the GenBank database under the accession numbers *VmD6PKL2* (MN053921), *VfD6PKL2* (MN053922), *VmSYT3* (MN053923), *VmBRXL4* (MN053925), *VmABCI15* (MN053927), *VmG6PD6* (MN053928) and *VmHLB1* (MN053929). The GenBank address is www.ncbi.nlm.nih.gov/genbank.

## Supplementary information

Supplementary Material
