# Supplementary material for: D6 protein kinase in root xylem benefiting resistance to Fusarium reveals infection and defense mechanisms in tung trees
Source: Hortic Res. 2021 Nov 1;8:240. doi: 10.1038/s41438-021-00656-2 (PMC8558330; doi:10.1038/s41438-021-00656-2)
Supplement: Supplementary file 1 — Supplementary Material [file 41438_2021_656_MOESM1_ESM.pdf]

Supporting Information

D6 protein kinase in root xylem benefiting the resistance to Fusarium reveals infection and defense mechanisms in tung tree

Zhang *et al*.

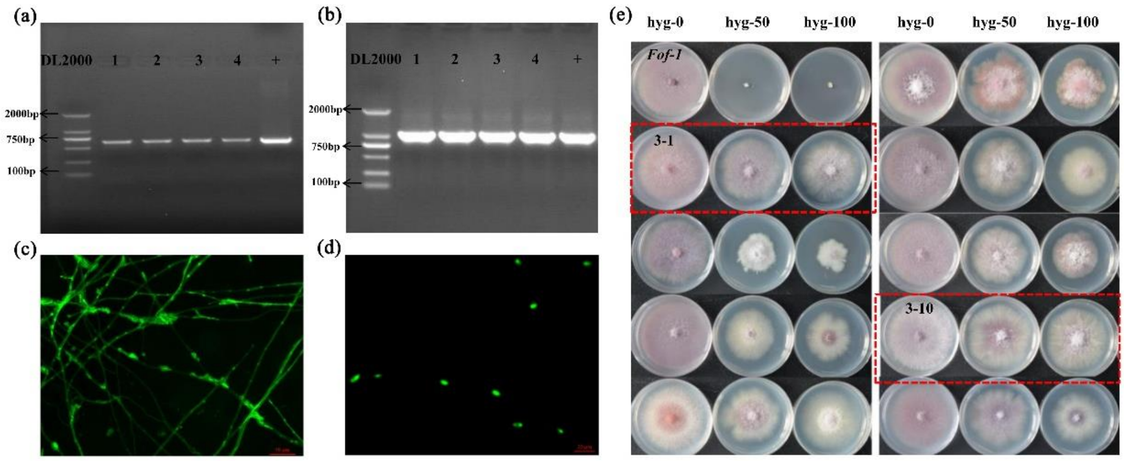

Fig. S1 ***A. tumefaciens*****-mediated transformation of** ***Fof-1.*** The expression of *GFP* (a) and *hph* (b) of positive transformants. DL2000 represents the DNA marker. Lanes 1, 2, 3 and 4 indicate the transformants, and lane + indicates the positive control. The fluorescence expression of mycelium (c) and conidium (d) of positive transformations. The scale bars are 50 μm and 20 μm, respectively. (e) After five successive transfers on PDA without hygromycin B, the phenotype of transformants cultured on PDA containing hygromycin B was assayed. Hyg-0, hyg-50 and hyg-100 represent the concentrations of hygromycin B of 0, 50, 100 $\mu g \cdot ml^{-1}$, respectively.

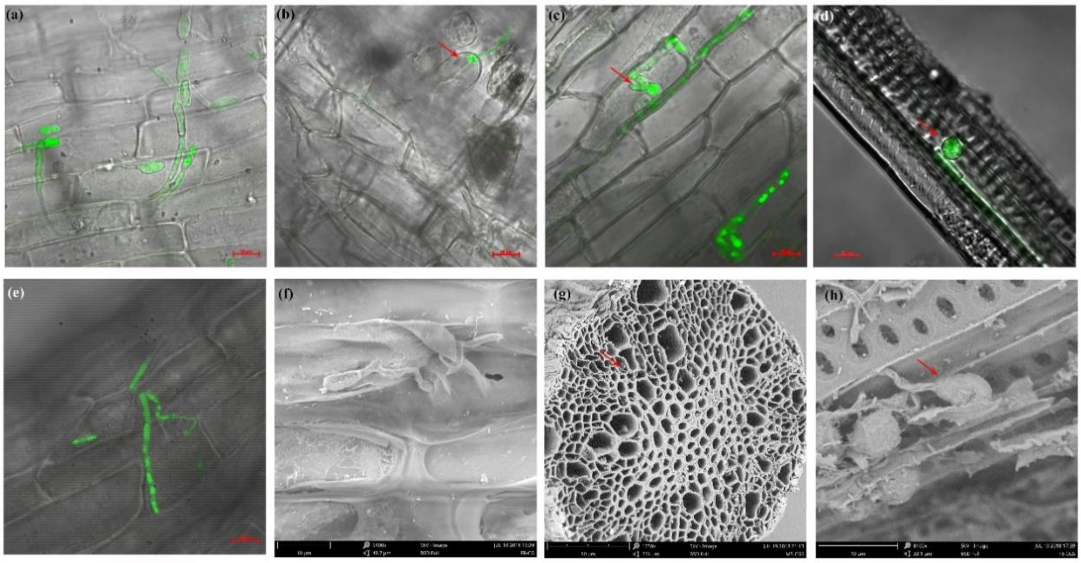

Fig. S2 Penetration site and specific infection structures of *Fof-1*. (a-b, f) Epidermis of lateral root of *V. fordii* at 1 dpi. (c) Cortex of lateral root of *V. fordii* at 3 dpi. (d, h) Xylem of lateral root of *V. fordii*. (e) Epidermis of lateral root of *V. montana* at 1 dpi. (g) Xylem of lateral root of *V. montana*. Bars, 10 μm.

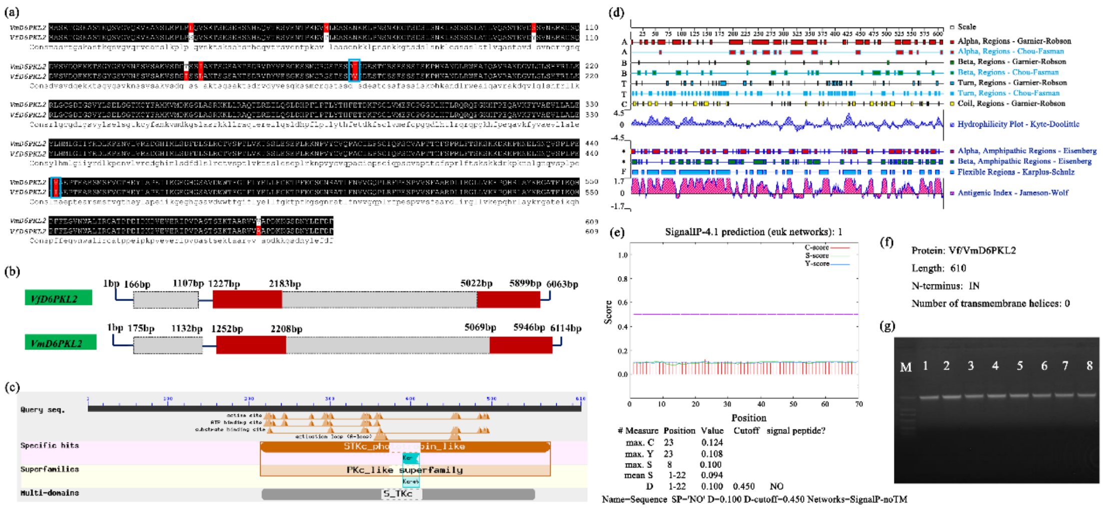

Fig. S3 Physicochemical and structural analysis of *VmD6PKL2* and *VfD6PKL2*. (a) The alignment of amino acids sequences between *VmD6PKL2* and *VfD6PKL2*. The amino acids residues on black background represent the consensus sequences, and the residues on red background represent the polymorphic sequences. (b) The structures of *VfD6PKL2* and *VmD6PKL2*. The red boxes indicate exons, the gray boxes indicate introns, and the black lines indicate 3′ and 5′ UTR. Numbers indicate lengths of sequences. (c) Analysis of the putative conserved domains of *Vm/VfD6PKL2*. (d) Hydrophobic analysis of *Vm/VfD6PKL2*. Twelve amino acid scales were used. The positions with positive values are hydrophobic, and those with negative values are hydrophilic. (e) Prediction of signal peptides of *Vm/VfD6PKL2*. The mean-score deviation was 0.1, which was less than the D-cutoff 0.45. (f) Prediction of transmembrane helices of *Vm/VfD6PKL2*. (g) Alternative splicing analysis of *VfD6PKL2* or *VmD6PKL2*. M represents the DL2000 DNA marker. Lanes 1, 2, 3, and 4, respectively, indicate the amplified sequence of *VmD6PKL2* at 0, 1, 8, and 14 dpi, and lanes 5, 6, 7, and 8 indicate the amplified sequence of *VfD6PKL2* at the four infection stages.

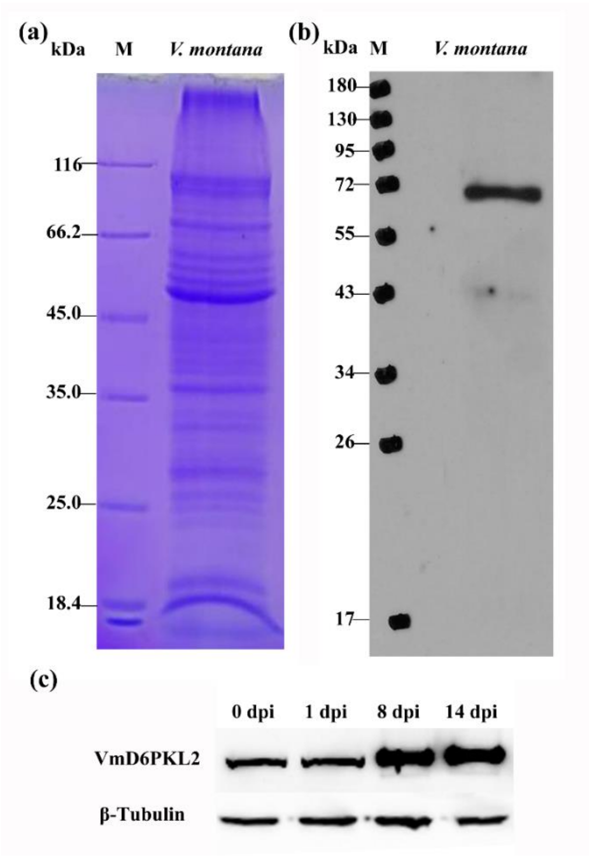

Fig. S4 Extraction of total protein of *V. montana* and detection of the VmD6PKL2. (a) The SDS-PAGE analysis of the total protein of *V. montana*. M indicated the protein marker; the sample quantity of the total protein was 5 μl and the concentration of the separation gel is 12%. (b) Detection of the VmD6PKL2 in total protein with anti-D6PKL2 antibody. The antibody was diluted 1: 5,000, and the gel was exposed for 2 min. (c) Expression analysis of VmD6PKL2 infected with the *Fof-1* at 0 dpi, 1 dpi, 8 dpi and 14 dpi with western blot analysis. β-Tubulin was used as internal control.

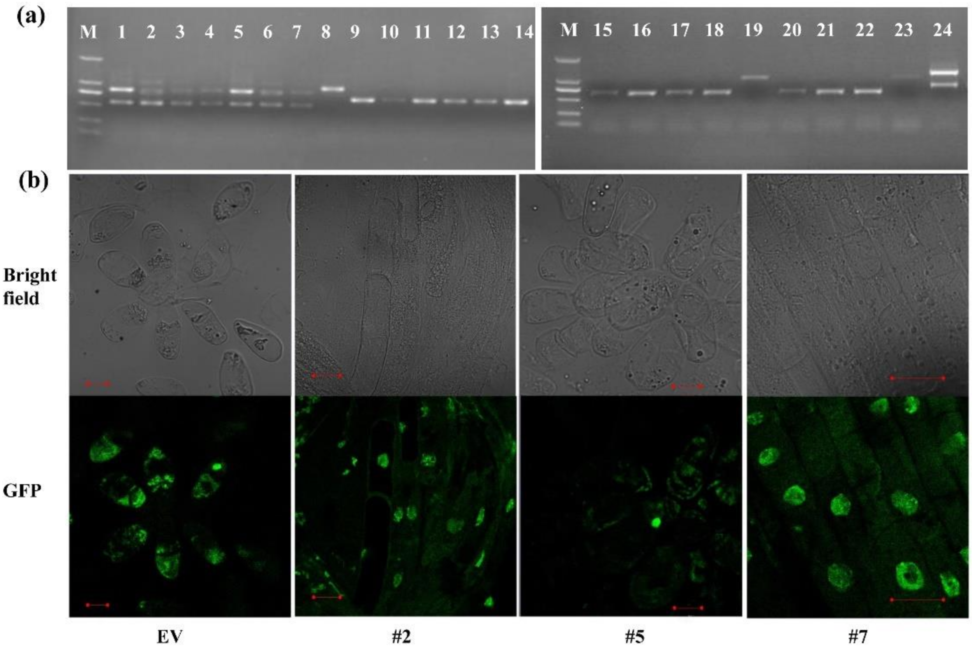

Fig. S5 Detection of mutant and transgenic *Arabidopsis*. (a) Verification of the homozygous T-DNA insertion mutants of *d6pkl2-1*, *d6pkl2-2* and *d6pkl2-3* using the three-primer method. M indicates the DL2000 DNA marker. Lanes 1-7 represent *d6pkl2-2*, and lane 8 represents the wild-type Col-0. Lanes 9-14 represent *d6pkl2-1*, and lanes 15-24 represent *d6pkl2-3*. (b) The expression of GFP fluorescence in transgenic *Arabidopsis*. EV and #2, #5, and #7 indicate the positive transgenic lines overexpressing empty vector and *VmD6PKL2*, respectively. GFP fluorescence was excited with 488 nm lasers and detected at 500-530 nm. Bars: 10 µm, 20 µm.

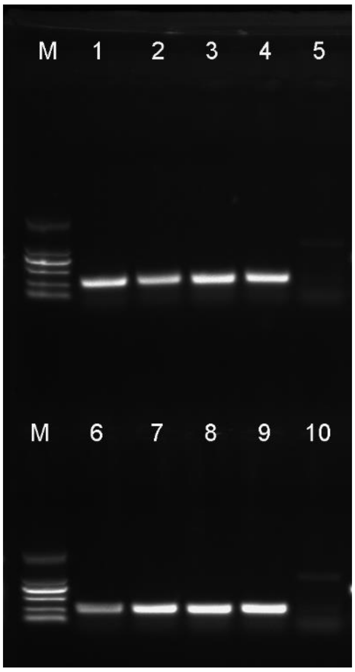

Fig. S6 Detection of transgenic tomatoes lines expressing *VmD6PKL2*. M1 indicates the DL2000 DNA marker. Lanes 1-4, and lanes 6-9 represent transgenic tomato lines using different specific primers of *VmD6PKL2*, as shown in Table S1. Lane 5 and 10 represent the wide-type tomatoes.

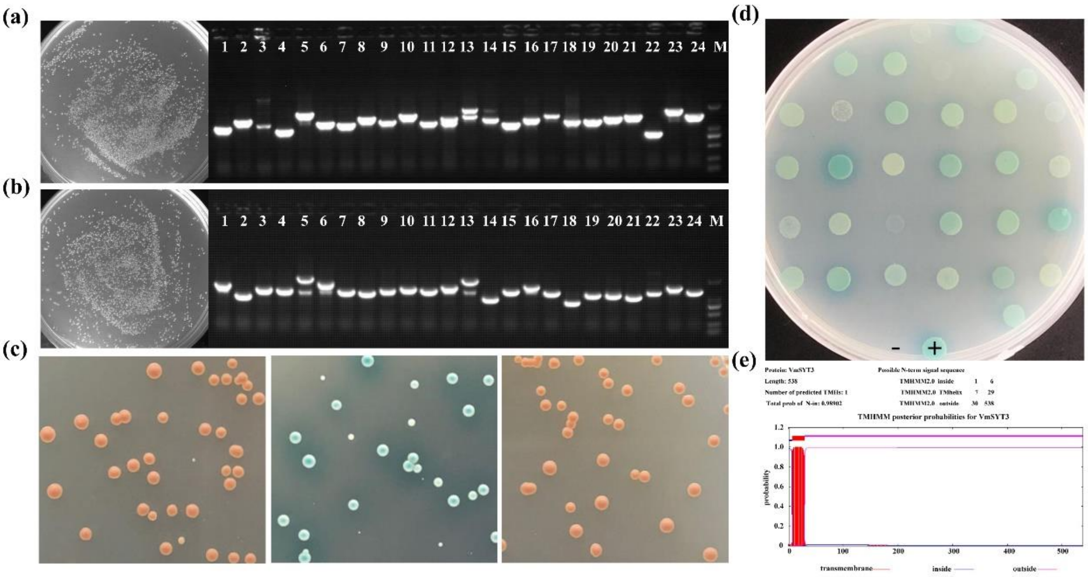

Fig. S7 Construction of cDNA library and yeast two-hybrid screening. (a-b) Calculation of CFU, recombination frequency and length of inserted fragments of uncut and secondary cDNA library. Lanes 1-24 represent the 24 randomly selected colonies, and M represents the DL2000 DNA marker. (c) Self-activation ability assay of VmD6PKL2. The negative control (left) and positive control (middle) grow on SD/-Leu-Trp/X-α-Gal medium, and the PGBKT7-*VmD6PKL2* grow on the SD/-Trp-X-α-Gal medium (right). (d) High-precision screening of 32 potential positive colonies with SD/-Ade-Trp-Leu-His/X-α-Gal/AbA medium. Colonies with blue color represent the positive transformants. (e) Prediction of transmembrane helices in VmSYT3.

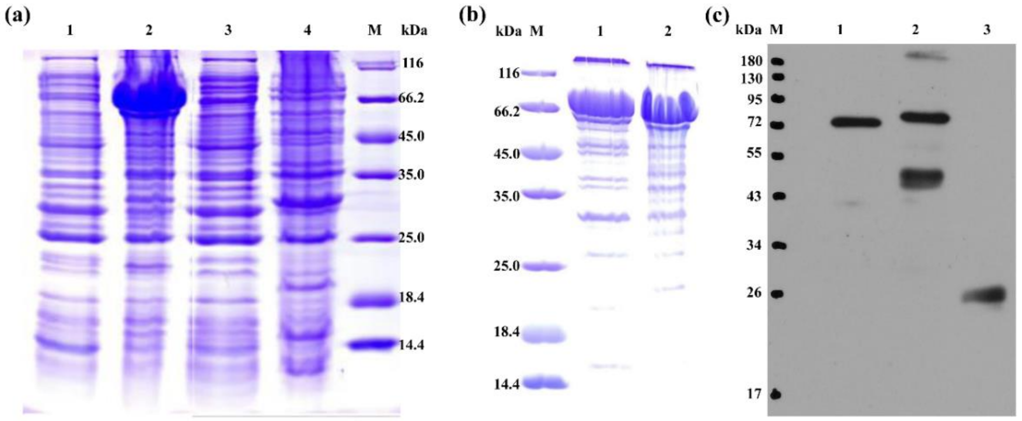

Fig. S8 Expression, purification and detection of GST-VmSYT3 and GST-VmBRXL4 recombinant proteins. (a) The supernatant and insoluble protein of the *E. coli* expressing GST-VmBRXL4 and GST-VmSYT3. Lane M indicates the protein marker, lines 1-2 respectively indicate the supernatant and insoluble protein of the *E. coli* expressing GST-VmBRXL4, and lines 3-4 respectively indicate the supernatant and insoluble protein of the *E. coli* expressing GST-VmSYT3. (b) SDS-PAGE analysis of the purified GST-VmSYT3 and GST-VmBRXL4 proteins. Lanes 1-2 indicate the purified GST-VmSYT3 and GST-VmBRXL4 protein, respectively. (c) Western blot analysis of the purified proteins with anti-GST antibody. Lanes 1-3 indicate the purified GST-VmBRXL4, GST-VmSYT3 and GST protein, respectively. The antibody was diluted 1: 50,000, and the gel was exposed for 2 min.

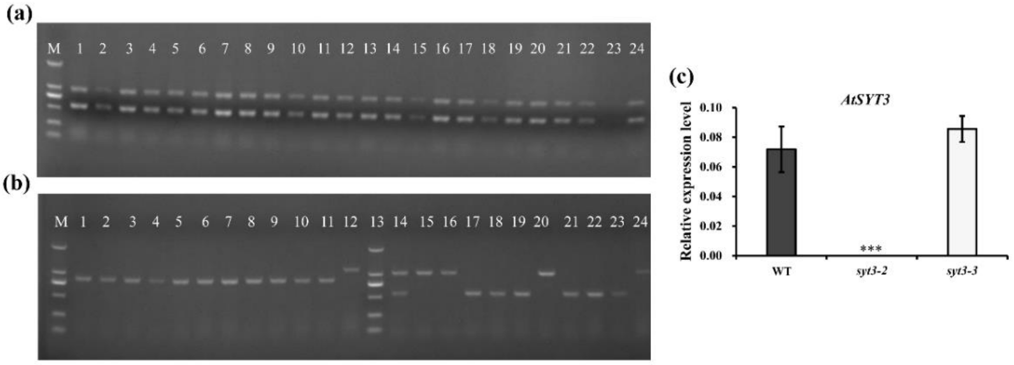

Fig. S9 Homozygosity verification of *syt3* mutants. M indicates the DL2000 DNA marker. (a) Lanes 1-24 represent *syt3-1*. (b) Lanes 1-11 represent *syt3-2*, lane 12 represents the wild-type Col-0, and lanes 14-24 represent *syt3-3*. (c) Relative expression levels of *AtSYT3* in wild-type and mutant *Arabidopsi*s. Expression level was analyzed using qRT-PCR in triplicate.

Table S1 Specific primers used in this study.

genetic name	Primer (5'-3')	length	application
LBb1.3	ATTTTGCCGATTTCGGAAC	19	confirmation of the homozygous T-DNA insertion mutants
d6pkl2-1-LP	CGGCAGATTTCACTAGAGTCG	21	
d6pkl2-1-RP	GTCATGGAGCTGAAGTCGAAG	21	
d6pkl2-2-LP	CTTCGCCTTTGATGATCTCTG	21	
d6pkl2-2-RP	AGTGACGAGAGTAGCTGCAGC	21	
d6pkl2-3-LP	AGTGTTGGGGTGAACTGACAG	21	
d6pkl2-3-RP	TGCTGCTTCAATTACATGCAC	21	
syt3-1-LP	AATGGCAACAGAAAATTCACC	21	
syt3-1-RP	AGTCTCAACTAGTGGCCTCGC	21	
syt3-2-LP	TAACGAGACCGATGAATGAGC	21	
syt3-2-RP	GAGGCCTGTTCTGTTTGTTTG	21	
syt3-3-LP	CGAACCCTTATGCTGTTGTTC	21	
syt3-3-RP	ATCCTCTGGTGGTTTCAAAGG	21	
VfD6PKL2-CDS-F	TCATCCTTTCTCTCTCTTCTCTCCACA	27	amplification of the ORF sequence of *VfD6PKL2* and *VmD6PKL2*
VfD6PKL2-CDS-R	ACAGGCAACAGCAAGCATACATTTC	25	
VmD6PKL2-CDS-F	ACACACTCTCCTCAAAGACCAAACC	25	
VmD6PKL2-CDS-R	TACCAATAAGTACACCACCCACCC	24	
VmSYT3-CDS-F2	TATTCATGCCATGTTCTTGATTTTC	25	amplification of the ORF sequence of *VmSYT3* and *VmBRXL4*
VmSYT3-CDS-R2	CCATTTTACCTTCATTCCATTTCTTTC	27	
VmBRXL4-CDS-F	TCTCTTTCCTATTTCTCTCCTCCAG	25	
VmBRXL4-CDS-R	ATACACAACCCGTTGCTCTTTACC	24	
VmD6PKL2-intron-F	TCTCCGCTTTCCCTTTTATCCTC	23	amplification of the intron sequence of *VfD6PKL2* and *VmD6PKL2*
VmD6PKL2-intron-R	CTTTATTTCTGTTGCCCCTCGCT	23	

VfD6PKL2-intron-F	TCTTTTTCTCTCAATCTCCGC	21	
VfD6PKL2-intron-R	ACTTCCATGACCTTCACCCTT	21	
*D6PKL2*AS-F12	TCTCTCAATCTCCGCTTTCCCTTT	24	splicing analysis of *VfD6PKL2* and *VmD6PKL2*
*D6PKL2*AS-R1	GTGTTGCTTTATTTCTGTTGCCCCT	25	
*D6PKL2*AS-R2	CCCTCGCTTGTATGCCAATCTATG	24	
GFP-VmD6PKL2-F	tcagcagtcgaagagcATGGCCTCGAGAACTGGCA	35	plasmid construction for overxpression of *VmD6PKL2 in Arabidopsis*
GFP-VmD6PKL2-R	ttagcgtgtgaagagcAAAGAAATCGAACTCCAGA	35	
GFP-F	CTGGTCGAGCTGGACGGCGACG	22	detection of the expression of GFP and hygromycin B in transformant *Fof-1*, *Arabidopsis* and tomatoes
GFP-R	CACGAACTCCAGCAGGACCATG	22	
HYG-F	ATGAAAAAGCCTGAACTCACCGCG	24	
HYG-R	CTATTTCTTTGCCCTCGGACGAG	23	
GFP-VmD6PKL2-F2	GGTACCTCTAGAGAAACCGGTGGATC	26	detection of the expression of *VmD6PKL2* in transformant *Arabidopsis* and tomatoes
GFP-VmD6PKL2-R2	TTTTACTGCTTGCTCAGGGAAATGCT	26	
M13-F	GTAAAACGACGGCCAG	16	yeast two-hybrid screening of VmD6PKL2
M13-R	CAGGAAACAGCTATGAC	17	
pGADT7-F (T7)	TAATACGACTCACTATAGGGCGAGCGCCGCCATG	34	
pGADT7-R (ADR)	GTGAACTTGCGGGGTTTTTCAGTATCTACGATT	33	
BD-VmD6PKL2-F	CGgaattcATGGCCTCGAGAACTGGCAGC	29	
BD-VmD6PKL2-R	CGggatccCTAAAAGAAATCGAACTCCAGAT	31	

pGEX-BRXL4-F	CCCTGGGATCCCCGGAATTCATGCTGACGTGTATAGCTCG	40	plasmid construction for GST-pull down analysis
pGEX-BRXL4-R	TCACGATGCGGCCGCTCGAGTTACAAGTATTGTTCGTGTA	40	
pGEX-SYT3-F	CCCTGGGATCCCCGGAATTCATGGACACCTTGCAAGATAT	40	
pGEX-SYT3-R	TCACGATGCGGCCGCTCGAGTCAAACTGTATCCCACCGTA	40	
q*EF1a*-F	GCCTGGTATGGTTGTGACCT	20	qPCR analysis of *D6PKL2 and SYT3* in *V. fordii*, *V. montana* and *Arabidopsis*
q*EF1a*-R	GGATCATCCTTGGAGTTGGA	20	
q*D6PKL2*-F	TCAAAGAGTGGAAGCAAACAGTC	23	
q*D6PKL2*-R	TACTCAACGAATCAGAAGTCCCT	23	
q*SYT3*-F	CATGGAACCAGCAATTAGGTGG	22	
q*SYT3*-R	GCAAAACAAGGAAAGGTAGGCA	22	
q*AtUBQ5*-F	GACGCTTCATCTCGTCC	17	qPCR analysis of *AtD6PKL2*, *AtSYT3*, GFP, and *VmD6PKL2GFP* in *Arabidopsis*
q*AtUBQ5*-R	CCACAGGTTGCGTTAG	16	
q*AtD6PKL2*-F2	CTCCCTATCCACTGTTCTTGA	21	
q*AtD6PKL2*-R2	ACTTAGCTTGGCACTTCCAC	20	
q*AtSYT3*-F1	GAACCATCAATCAAATGGGC	20	
q*AtSYT3*-R1	AGGCTTCAAAGCTACACGCA	20	
qAt-*GFP*-F1	CACTACCAGCAGAACACC	18	
qAt-*GFP*-R1	AACTCCAGCAGGACCATG	18	
qAt-*VmD6PKL2GFP*-F2	CTCGCGTTGTAGTTGCTCCTG	21	
qAt-*VmD6PKL2GFP*-R2	CCGTTTACGTCGCCGTCC	18	
